# Hepatitis A vaccination coverage survey in 24-month-old children living in Brazilian capitals, 2020

**DOI:** 10.1590/S2237-96222024v33e20231162.en

**Published:** 2024-10-21

**Authors:** Winny Éveny Alves Moura, Karlla Antonieta Amorim Caetano, Juliana de Oliveira Roque e Lima, Lays Rosa Campos, Grazielle Rosa da Costa e Silva, José Cássio de Moraes, Ana Paula França, Carla Magda Allan Santos Domingues, Maria da Gloria Lima Cruz Teixeira, Sheila Araújo Teles, ICV 2020 Group, Adriana Ilha da Silva, Alberto Novaes Ramos, Ana Paula França, Andrea de Nazaré Marvão Oliveira, Antonio Fernando Boing, Carla Magda Allan Santos Domingues, Consuelo Silva de Oliveira, Ethel Leonor Noia Maciel, Ione Aquemi Guibu, Isabelle Ribeiro Barbosa Mirabal, Jaqueline Caracas Barbosa, Jaqueline Costa Lima, José Cássio de Moraes, Karin Regina Luhm, Karlla Antonieta Amorim Caetano, Luisa Helena de Oliveira Lima, Maria Bernadete de Cerqueira Antunes, Maria da Gloria Teixeira, Maria Denise de Castro Teixeira, Maria Fernanda de Sousa Oliveira Borges, Rejane Christine de Sousa Queiroz, Ricardo Queiroz Gurgel, Rita Barradas Barata, Roberta Nogueira Calandrini de Azevedo, Sandra Maria do Valle Leone de Oliveira, Sheila Araújo Teles, Silvana Granado Nogueira da Gama, Sotero Serrate Mengue, Taynãna César Simões, Valdir Nascimento, Wildo Navegantes de Araújo

**Affiliations:** 1 Universidade Federal de Goiás, Faculdade de Enfermagem, Goiânia, GO, Brazil; 2 Santa Casa de São Paulo, Faculdade de Ciências Médicas, São Paulo, SP, Brazil; 3 Organização Pan-Americana da Saúde, Brasília, DF, Brazil; 4 Universidade Federal da Bahia, Instituto de Saúde Coletiva, Salvador, BA, Brazil; Universidade Federal do Espírito Santo, Vitória, ES, Brazil; Universidade Federal do Ceará, Departamento de Saúde Comunitária, Fortaleza, CE, Brazil; Faculdade Ciências Médicas Santa Casa de São Paulo, São Paulo, SP, Brazil; Secretaria de Estado da Saúde do Amapá, Macapá, AP, Brazil; Universidade Federal de Santa Catarina, SC, Brazil; Organização Pan-Americana da Saúde, Brasília, DF, Brazil; Instituto Evandro Chagas, Belém, PA, Brazil; Faculdade de Ciências Médicas Santa Casa de São Paulo, Departamento de Saúde Coletiva, São Paulo, SP, Brazil; Universidade Federal do Rio Grande do Norte, Natal, RN, Brazil; Universidade Federal do Ceará, Departamento de Saúde Comunitária, Fortaleza, CE, Brazil; Universidade Federal de Mato Grosso, Cuiabá, MT, Brazil; Universidade Federal do Paraná, Curitiba, PR, Brazil; Universidade Federal de Goiás, Goiânia, GO, Brazil; Universidade Federal do Piauí, Teresina, PI, Brazil; Universidade de Pernambuco, Faculdade de Ciências Médicas, Pernambuco, PE, Brazil; Instituto de Saúde Coletiva, Universidade Federal da Bahia, Salvador, BA, Brazil; Secretaria de Estado da Saúde de Alagoas, Maceió, AL, Brazil; Universidade Federal do Acre, Rio Branco, AC, Brazil; Universidade Federal do Maranhão, Departamento de Saúde Pública, São Luís, MA, Brazil; Universidade Federal de Sergipe, Aracaju, SE, Brazil; Secretaria Municipal de Saúde, Boa Vista, RR, Brazil; Fundação Oswaldo Cruz, Mato Grosso do Sul, Campo Grande, MS, Brazil; Fundação Oswaldo Cruz, Escola Nacional de Saúde Pública Sergio Arouca, Rio de Janeiro, RJ, Brazil; Universidade Federal do Rio Grande do Sul, Porto Alegre, RS, Brazil; Fundação Oswaldo Cruz, Instituto de Pesquisa René Rachou, Belo Horizonte, MG, Brazil; Secretaria de Desenvolvimento Ambiental de Rondônia, Porto Velho, RO, Brazil; Universidade de Brasília, Brasília, DF, Brazil

**Keywords:** Cobertura de Vacunación, Programas de Inmunización, Vacunas Contra la Hepatitis A, Niño, Factores Socioeconómicos, Encuestas Epidemiológicas, Vaccination Coverage, Immunization Programs, Hepatitis A Vaccines, Child, Socioeconomic Factors, Epidemiological Surveys

## Abstract

**Objective:**

To estimate hepatitis A vaccination coverage in 24-month-old children and identify factors associated with non-vaccination.

**Methods:**

This was a survey involving a sample stratified by socioeconomic strata in capital cities (2020-2022), with coverage estimates and 95% confidence intervals (95%CI), the factor analysis was performed using the prevalence ratio (PR) by means of Poisson regression.

**Results:**

Among 31,001 children, hepatitis A coverage was 88.1% (95%CI 86.8;89.2). Regarding socioeconomic strata (A/B), the variable immigrant parents/guardians was associated with non-vaccination (PR = 1.91; 95%CI 1.09;3.37); in strata C/D, children of Asian race/skin color (PR = 4.69; 95%CI 2.30;9.57), fourth-born child or later (PR = 1.68; 95%CI 1.06;2 .66), not attending daycare/nursery (PR = 1.67; 95%CI 1.24;2.24) and mother with paid work (PR = 1.42; 95%CI 1.16;1.74) were associated with non-vaccination.

**Conclusion:**

Hepatitis A coverage was below the target (95%), suggesting that specificities of social strata should be taken into consideration.

## Study contributions

**Main results:**

Hepatitis A vaccination coverage was 88%. Non-vaccination was greater in children with immigrant guardians (strata A/B); of Asian race/skin color, fourth-born child or later, those not attending daycare/nursery and mother with paid work (C/D strata).

**Implications for services:**

The results of this study contributed to the Ministry of Health and Health Departments in monitoring vaccination coverage and identifying factors that may negatively impact hepatitis A vaccination coverage.

**Perspectives:**

Further research is needed on the impact of migration on hepatitis A vaccination and vaccination in general. Health managers should be attentive to the different factors affecting vaccination among social strata.

## RESUMEN

**Objetivo:**

Estimar cobertura de vacunación contra hepatitis A en niños de 24 meses y identificar factores associados a falta de vacunación.

**Métodos:**

Encuesta en muestra estratificada por estratos socioeconómicos en capitales (2020-2022), con estimación de cobertura e intervalos de confianza del 95% (IC_95_%) y análisis de factores por la razón de prevalencia (RP) mediante regresión de Poisson.

**Resultados:**

En los 31.001 niños, la cobertura de hepatitis A fue del 88,1% (IC_95_% 86,8%; 89,2%). Em estratos socioeconómicos (A/B), variable padres/tutores inmigrantes se asoció con la ausência de vacunación (RP = 1,91; IC_95_% 1,09;3,37); en estratos C/D, variables niño amarillo (RP = 4,69; IC_95_% 2,30;9,57), cuarto orden de nacimiento o más (RP=1,68; IC_95_% 1,06;2,66), no asistir a guardería/jardín de infancia (RP = 1,67; IC_95_% 1,24;2,24) y madre con trabajo remunerado (RP = 1,42; IC_95_% 1,16;1,74) se asociaron la ausência de vacunación.

**Conclusión:**

Cobertura de la hepatitis A por debajo del objetivo (95%), sugiriendo considerar las especificidades de los estratos sociales.

## INTRODUCTION

Globally, it is estimated that more than 100 million cases of hepatitis A occur annually, resulting in 15,000 to 30,000 deaths due to the disease mainly in developing countries, where access to safer water and sanitation is inadequate.^1^ In Brazil, improvements in the population’s living conditions, especially in the early decades of the 21^st^ century, resulted in the epidemiological transition of hepatitis A, with low endemicity observed in the most developed regions, such as the South and Southeast regions, and intermediate endemicity in the least developed regions, such as the Midwest, North and Northeast regions.^2^

Hepatitis A, caused by the hepatitis A virus (HAV), RNA virus of the *Picornaviridae family*, genus Hepatovirus^1^ is transmitted primarily via the fecal-oral route. In general, it causes self-limiting acute inflammatory liver disease and rarely progresses to the fulminant form. In early childhood, the infection is usually asymptomatic, with the risk of symptoms increasing with age, including fever, malaise, fatigue, loss of appetite, diarrhea, nausea, abdominal discomfort, anorexia, myalgia, headache, arthralgia and jaundice. On the other hand, more than 70% of children over 5 years of age and adults are symptomatic, requiring rest and hospitalization. Fulminant hepatitis A is a rare event, and occurs more frequently in older adults.^3^

Hepatitis A vaccines are safe and effective. Formaldehyde-inactivated hepatitis A vaccines are licensed for use in children aged 12 months and older, administered intramuscularly, with two-dose schedule and a minimum six-month interval between doses.^1^ However, studies in developing countries have shown the effectiveness of these vaccines with a single-dose schedule,^4,5^ leading the World Health Organization to recommend both schedules (one or two doses) for children.^6^ Thus, taking into consideration Brazil’s new endemic profile, and after cost-effectiveness analyses, the National Committee for Health Technology Incorporation recommended in 2012 the inclusion of the hepatitis A vaccine in the routine childhood vaccination schedule.^7^ In 2014, the National Immunization Program (*Programa Nacional de Imunizações* - PNI) introduced this vaccine into the childhood schedule, with a single-dose schedule, at 15 months of age.^8^

A study using secondary data, which evaluated the incidence of hepatitis A and hepatitis A vaccination coverage in Brazil, five years after its implementation in the PNI (2014-2018), found that vaccination coverage had its worst performance in 2014 (60 .13%) and its best performance in the following year (97.07%), with negative variations in subsequent years: 71.58% (2016), 82.7% (2017) and 76.72% (2018). Despite this variation, as observed in other countries, there was a reduction in the incidence of hepatitis A.^9^

Between 2014 and 2022, there was a decrease of more than 95% in the incidence of hepatitis A in children under 10 years of age in Brazil,^10^ reinforcing the need to achieve the target of 95% hepatitis A vaccine coverage, recommended by the Ministry of Health,^11^ for reducing incidence and controlling the disease. Therefore, periodic assessment of vaccination coverage is essential in order to identify any service shortcomings that need to be addressed, with population-based surveys being the most reliable sources of information. The objective of this study was to estimate hepatitis A vaccination coverage in 24-month-old children and identify factors associated with non-vaccination.

## METHODS

This was a population-based household epidemiological survey that is part of the National Vaccination Coverage Survey (*Inquérito Nacional de Cobertura Vacinal* - INCV),^12^ conducted in the five regions of Brazil. This segment of the study evaluated the coverage of the hepatitis A vaccine in children born alive in 2017 and 2018, residing in 26 capitals and the Federal District, according to records from the Live Birth Information System (*Sistema de Informações sobre Nascidos Vivos* - SINASC).

Data collection took place between September 2020 and March 2022, respecting physical distancing due to the COVID-19 pandemic.

Detailed information on sample calculation and data collection was described in a methodological study by Barata et al.^12^ In summary, the sample size per survey was calculated considering the following parameters: a design effect of 1.4; an estimated prevalence of vaccination coverage of 70%; an estimation error of 5%; and a z of 1.96 for a 95% confidence interval. Thus, 452 children were needed per survey. Taking into consideration the heterogeneity of vaccination coverage in the capitals, one to four surveys were conducted in each city, depending on the number of live births recorded in SINASC in 2017 and 2018. For comparison purposes, the sample size was divided in order to ensure the same number of children per socioeconomic stratum. 

For the definition of socioeconomic strata, all urban census sectors of each city were used, according to information from the 2010 demographic census.^13^ Thus, in cities where only one survey was conducted, such as Porto Velho, the capital city of the state of Rondônia, the number of children per stratum was 113. In cities such as São Paulo, the capital of the state of São Paulo, where four surveys were conducted, the number of children per stratum was 452. The classification of the sectors took into account the average income of those who were the heads of household, the proportion of literate household heads and the proportion of household heads with income greater than or equal to 20 minimum wages. The social strata were classified from A to D, based on the level of family consumption, with cutoff points established by the Brazilian Association of Research Companies,^14^ Brazil criteria: stratum A, high (42 points and more); stratum B, medium (27 to 41 points); stratum C, low (16 to 26 points); and stratum D, very low (< 16 points).

Two collection instruments were used: a structured questionnaire with closed ended-questions and a photograph of the vaccination booklet. The questionnaire was comprised of blocks of questions, which for this study included the child’s sociodemographic data; mother’s reproductive and sociodemographic data; household and family income data; and data transcribed from the vaccination booklet (valid doses administered and date of administration) from PNI and the private sector. Valid doses (between 12 and 24 months) were those administered within the correct interval and/or around the expected date. 

Vaccination coverage was defined as the percentage of children who received the hepatitis A vaccine, calculated according to valid doses. For the purposes of this study, the numerator was the number of children born in 2017 and 2018 who received the hepatitis A vaccine, and the denominator was the total number of children born alive during the same period in the urban areas of Brazilian capitals.

The analysis of the characteristics associated with non-vaccination against hepatitis A in the social strata included key variables for understanding the underlying factors. The variables associated with non-vaccination against hepatitis A related to children were sex (female, male), birth order (the order a child is born in relation to their siblings), child’s race/skin color (White, Black, mixed-race, Asian and Indigenous), attending daycare/school (yes; no) and being a beneficiary of the Bolsa Família program (yes; no). The variable associated with non-vaccination against hepatitis A related to families was family income (in BRL: up to BRL 1,000.00; from BRL 1,001.00 to BRL 3,000.00; from BRL 3,001.00 to R$8,000.00; greater than BRL 8,000.00). The maternal variables or those related to the guardian were schooling (complete elementary education or incomplete elementary education; complete middle school or incomplete high school; complete high school or incomplete higher education; complete higher education or above), age at child’s birth (< 20 years; 20 - 34 years old; ≥ 35 years old), race/skin color (White, Black, mixed-race, Asian and Indigenous), paid work (yes; no), immigrant status of the guardian (no; yes) and mother’s marital status (with a stable partner; without a stable partner).

### Data analysis

As described in detail by Barata et al.,^12^ since this is a complex sample, sample weights were calculated for each household interviewed, in order to allow for unbiased estimation of the population’s parameters of interest. This procedure occurred in two stages: initially, basic sampling weights were obtained (inverse of the probabilities of inclusion of the households interviewed), subsequently, these weights were calibrated for known population totals. In order to do this, the most relevant data such as children’s vaccination and possible reasons for non-vaccination were used. The software Stata version 17, survey data analysis module, was used for data analysis. Overall hepatitis A vaccination coverage, by region of Brazil and by social stratum, was estimated with their respective 95% confidence intervals (95%CI). In order to identify potential explanatory variables for non-vaccination against hepatitis A, two subgroups were considered for analysis: strata A/B and strata C/D. Initially, a bivariate analysis was performed to identify potential variables associated with non-vaccination against hepatitis A. Subsequently, variables with a p-value < 0.20 were included in multivariate Poisson regression models. A-Link Test was used to evaluate the quality of the final models. P-values < 0.05 were considered statistically significant.

### Ethical aspects

The survey was approved by the Human Research Ethics Committees of the Instituto de Saúde Coletiva da Universidade Federal da Bahia (opinion 3,366,818, on 04/06/2019, Certificate of Submission for Ethical Appraisal (CAAE) 4306919.5.0000.5030); and the Irmandade da Santa Casa de São Paulo (opinion 4,380,019, 04/11/2020, CAAE 39412020.0.0000.5479). Before starting the study, local coordinators informed state and municipal immunization authorities about the research. Interviewees (parents or guardians) gave written consent and allowed their vaccination booklets to be photographed. Data collection took place only after obtaining the guardians’ authorization, ensuring confidentiality. The database used excluded identifying information.^12^

## RESULTS

**Figure 1:**
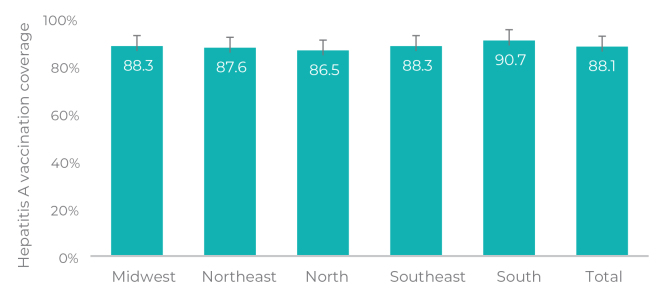
Hepatitis A vaccination coverage in 24-month-old children (n = 31,001), living in capital cities, according to the region of Brazil, 2020-2021

A total of 33,032 children were recruited and eligible for the study and 31,001 children took part, representing a loss of 6.15% of the initially recruited sample. Losses occurred due to refusals, after three unsuccessful attempts to visit the interviewer at different times and on different days, and a number of children lower than expected in the randomly selected clusters. Figure 1 shows hepatitis A vaccine coverage in the cohort of children born in 2017 and 2018, in Brazil and its regions. The overall hepatitis A vaccine coverage was 88.1% (95%CI 86.8;89.2), ranging from 86.5% (95%CI 84.0;88.7), in the North region, to 90 .7% (95%CI 87.7;93.0), in the South region.

**Figure 2:**
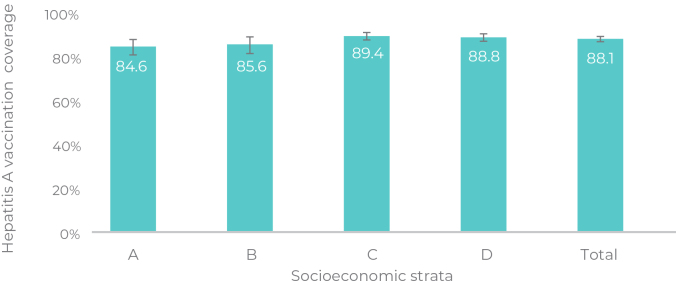
Hepatitis A vaccination coverage in 24-month-old children (n = 31,001), living in capital cities, according to socioeconomic stratum, Brazil, 2020-202

Figure 2 shows hepatitis A vaccine coverage in Brazil, according to socioeconomic stratum. Strata C (89.4%) and D (88.8%) had higher coverage than strata A (84.6%) and B (85.6%) (p = 0.032).

In strata A and B, children whose guardians were foreign immigrants had a 1.91 times higher prevalence of non-vaccination when compared to those who were in the care of Brazilian guardians. On the other hand, in Black and mixed-race mothers’ children non-vaccination was 66% and 33% lower, respectively, when compared to White mothers’ children (Table 1).

**Table 1:** Frequency, crude and adjusted prevalence ratio (PR) and 95% confidence interval (95%CI) for non-vaccination against hepatitis A in 24-month-old Brazilian children (n = 31,001) and their mothers in social strata A/B, Brazil, 2020-2021

Variable	%	Yes (%)	PR (95%CI)^a^	p-value	Adjusted PR (95%CI )	p-value
**Child’s sex**						
Male	14.8	85.2	1.00			
Female	14.9	85.1	1.01 (0.74;1.36)	0.966		
**Birth order**						
First-born	13.4	86.6	1.00		1.00	
Second-born	16.7	83.3	1.25 (0.88;1.76)		1.16 (0.79;1.70)	
Third-born	15.1	84.9	1.13 (0.71;1.80)	0.159	1.14 (0.78;1.67)	0.115
Fourth-born or more	19.1	80.9	1.43 (0.83;2.46)		1.84 (1.06;3.18)	
**Child’s race/skin color**						
White	15.5	84.5	1.00			
Black	11.7	88.3	0.75 (0.44;1.28)			
Mixed-race	13.8	86.2	0.89 (0.68;1.17)	0.286		
Asian	7.0	93.0	0.45 (0.13;1.55)			
Indigenous	21.3	78.7	1.37 (0.59;3.20)			
**Attends daycare/school**						
Yes	13.5	86.5	1.00			
No	16.3	83.7	1.21 (0.90;1.61)	0.199		
Up to 1,000.00	13.8	86.2	1.00			
1,001.00 - 3,000.00	16.9	83.1	1.23 (0.87;1.73)			
3,001.00 - 8,000.00	10.0	90.0	0.72 (0.45;1.18)	0.100		
> 8,000.00	11.1	88.9	0.81 (0.49;1.31)			
**Beneficiary of Bolsa Família Program**						
Yes	14.1	85.9	1.00			
No	14.9	85.1	1.06 (0.78;1.45)	0.712		
**Mother’s schooling (years)**						
≤ 8	18.3	81.7	1.00			
9 - 12	14.8	85.2	0.81 (0.44;1.49)			
13 - 15	11.6	88.4	0.64 (0.36;1.12)	0.647		
≥ 16	15.2	84.8	0.83 (0.47;1.45)			
**Mother’s age (years) at child’s birth**						
< 20	6.7	93.3	1.00			
20 to 34	14.8	85.2	2.20 (0.99;4.90)	0.718		
35 or more	14.9	85.1	2.22 (0.99;4.97)			
**Mother’s race/skin color**						
White	16.7	83.3	1.00		1.00	
Black	6.8	93.2	0.40 (0.25;0.67)		0.34 (0.19;0.62)	
Mixed-race	12.0	88.0	0.72 (0.53;0.98)	0.012	0.67 (0.48;0.95)	0.045
Asian	6.4	93.6	0.38 (0.14;1.02)		0.81 (0.30;2.16)	
Indigenous	12.9	87.1	0.77 (0.28-2.11)		0.65 (0.23-1.81)	
**Mother has paid work**						
Yes	14.7	85.3	1.00			
No	14.2	85.8	0.97 (0.67;1.40)	0.855		
**Immigrant guardian**						
No	14.6	85.4	1.00		1.00	
Yes	36.5	63.5	2.49 (1.24;4.99)	0.010	1.91 (1.09;3.37)	0.037
**Marital status**						
With a partner	14.1	85.9	1.00			
Without a partner	15.0	85.0	1.06 (0.76;1.50)	0.719		

Multivariable analysis of data from children in strata C and D revealed that children of Asian race/skin color showed nearly five times higher prevalence of non-vaccination against hepatitis A when compared to those of White race/skin color. It could be seen that as the birth order increased, such as the second born-child (RP = 1.31; 95%CI 1.05;1.64) , the third-born child (RP = 1.54; 95%CI 1 .11;2.13) and the fourth-born child or later (RP = 1.68; 95%CI 1.06;2.66), the prevalence of non-vaccination was higher when compared to first-born children. Children who did not attend daycare/nursery and those whose mothers had paid work showed increased prevalence of 1.67 and 1.42, respectively, when compared to those who did not have such characteristics (Table 2).

**Table 2:** Frequency, crude and adjusted prevalence ratio (PR) and 95% confidence interval (95%CI) for non-vaccination against hepatitis A in 24-month-old Brazilian children (n = 31,001), living in 26 capitals and in the Federal District, and their mothers, in social strata C/D, Brazil, 2020-2021

Variable	%	Yes (%)	PR (95%CI ) ^a^	p-value	Adjusted PR (95%CI )	p-value
**Child’s sex**						
Male	10.7	89.3	1.00			
Female	11.4	88.6	1.01 (0.86;1.32)	0.574		
Birth order						
First-born	9.5	90.5	1.00		1.00	
Second-born	10.1	89.1	1.14 (0.90;1.44)		1.31 (1.05;1.64)	
Third-born	12.3	87.7	1.29 (0.94;1.76)	0.002	1.54 (1.11; 2.13)	0.003
Fourth-born or more	16.9	83.1	1.77 (1.22;2.56)		1.68 (1.06;2.66)	
**Child’s race/skin color**						
White	9.5	90.5	1.00		1.00	
Black	11.6	88.4	1.22 (0.87;1.70)		1.14 (0.79;1.65)	
Mixed-race	12.2	87.8	1.28 (1.03;1.59)	0.007	1.03 (0.81;1.31)	
Asian	35.3	64.7	3.70 (1.87;7.32)		4.69 (2.30;9.57)	0.371
Indigenous	7.7	92.3	0.81 (0.28;2.34)		0.57 (0.20;1.65)	
**Attends daycare/school**						
Yes	9.1	90.9	1.00		1.00	
No	12.8	87.2	1.40 (1.09;1.79)	0.008	1.67 (1.24;2.24)	0.001
**Family income (BRL)**						
Up to 1,000.00	12.2	87.8	1.00			
1,001.00 - 3,000.00	10.1	89.9	0.82 (0.64;1.07)			
3,001.00 - 8,000.00	8.4	91.6	0.69 (0.48;0.99)	0.003		
> 8,000.00	6.1	93.9	0.50 (0.30;0.83)			
**Beneficiary of Bolsa Família Program**						
Yes	9.9	90.1	1.00			
No	11.4	88.6	1.15 (0.92;1.44)	0.214		
**Mother’s schooling (years)**						
≤ 8	13.0	87.0	1.00			
9 - 12	13.2	86.8	1.01 (0.74;1.39)			
13 - 15	10.0	90.0	0.77 (0.54;1.10)	0.010		
≥ 16	8.3	91.7	0.64 (0.41;0.98)			
**Mother’s age (years) at child’s birth**						
< 20 years	10.8	89.2	1.00			
20 to 34 years old	12.4	87.6	1.15 (0.73;1.81)	0.009		
35 years or older	8.8	91.2	0.81 (0.49;1.34)			
**Mother’s race/skin color**						
White	8.7	91.3	1.00		1.00	
Black	11.7	88.3	0.40 (0.25;0.67)		1.34 (0.97;1.85)	
Mixed-race	12.0	88.0	0.72 (0.53;0.98)		1.37 (1.07;1.76)	
Asian	13.5	86.5	0.38 (0.14;1.02)	0.012	1.55 (0.59;4.08)	
Indigenous	10.1	89.9	0.77 (0.28-2.11)		1.16 (0.46;2.91)	
**Mother has paid work**						
Yes	11.9	88.1	1.28 (1.05;1.55)	0.012	1.42 (1.16;1.74)	0.001
No	9.3	90.7	1.00		1.00	
**Immigrant guardian**						
No	11.0	89.0	1.00			
Yes	17.9	82.1	1.63 (0.87;3.08)	0.128		
**Marital status**						
With a partner	10.4	89.6	1.00			
Without a partner	11.1	88.9	1.07 (0.84;1.35)	0.576		

## DISCUSSION

This investigation represents the first primary data on hepatitis A vaccination coverage from a population-based study, with analysis stratified by regions of Brazil and social strata, in addition to identifying factors associated with the non-vaccination. All regions of the country showed hepatitis A vaccination coverage below the target set by the Ministry of Health, with the highest socioeconomic strata showing the lowest coverage rates. 

Some limitations should be taken into consideration when interpreting the results presented. The study was conducted in urban areas and may not represent children from the entire country, although it represents a significant portion of the cohort studied. The 2010 Demographic Census was used to define socioeconomic strata, and urban changes over the decade may have occurred, but household-level sociodemographic data helped to mitigate this limitation. There was a high proportion of mothers aged 35 years and older with complete higher education, suggesting selection bias. In this study, maternal age and schooling were not associated with non-vaccination against hepatitis A in multiple analyses, suggesting a low impact on the results. Despite the limitations, this study included all regions of the country with a robust sample size and methodological rigor.

The estimated hepatitis A vaccination coverage observed in this study was above historical coverage averages from previous years, suggesting underreporting of data in the PNI Information System.^9^ Although regional variations were observed, the differences were not statistically significant, even though these vaccination coverages were below the desirable target of 95% in all regions. Notably, the North region, considered a high endemicity area for hepatitis A,^2^ showed the lowest vaccination coverage for this vaccine.

The decline in vaccination coverage in Brazil is multifactorial, including changes in the vaccine registration system, reduced investment in the health sector and the rise of the anti-vaccine movement supported by misinformation dissemination.^15^ The current scenario has been impacted by occasional vaccine shortages due to production difficulties, and operational challenges that have affected the adequate implementation of vaccination programs.^16^

Socioeconomic strata A/B, more economically advantaged, showed lower hepatitis A vaccination coverage than strata C/D, less advantaged. It is believed that the PNI’s high vaccination coverage until the mid-2010s reduced/eliminated several childhood infections, which may have led to a false sense of security among parents regarding their children’s health.^17^ Increased adherence to the anti-vaccine movement among the most privileged classes has contributed to the reduction in vaccination rates within these strata.^18^

The increase in vaccination coverage in the least privileged classes can be attributed mainly to the comprehensive work of the Family Health Strategy (*Estratégia Saúde da Família* ESF) and easier access to health facilities. This initiative has significantly expanded the availability of preventive measures, focusing on the most vulnerable communities.^17^ ESF teams play an active role in supporting families, monitoring children’s health, providing guidance on the importance of immunization and ensuring adherence to the vaccination schedule.^19^ The Bolsa Família program, a fundamental income transfer strategy, plays a pivotal role in promoting child health and ensuring vaccination, by establishing health conditions that include updating the vaccination schedule.^20^

The impact of possible sociodemographic characteristics on low hepatitis A vaccination coverage was analyzed, considering both socioeconomic strata A/B and C/D.

For strata A/B, the child’s race/skin color did not influence hepatitis A vaccination coverage, but the mother’s race/skin color played a protective role. Black and mixed-race mothers’ children had 66% and 33% lower prevalence of non-vaccination, respectively, when compared to those whose mothers were of White race/skin color. However, it is worth highlighting that within these strata, there is a difference in income distribution between families of Black and mixed-race mothers’ children and White mothers’ children. A smaller proportion of families with mothers of Black and mixed race/skin color (13.6%) had an income over BRL 8,000.00 compared to families with White mothers (44.8%) (data not shown), corroborating the findings of better vaccination coverage among lower-income families.

It could be seen in strata A/B that being a child in the care of immigrants was a factor associated with non-vaccination against hepatitis A. This reluctance towards vaccination among immigrants can be attributed to several factors, such as fear and misinformation about possible side effects of vaccines, lack of knowledge about diseases, distrust in the health systems of host countries, language barriers and the influence of beliefs and media from their home countries.^21^

In line with the findings in strata A/B, migration issues also appear to affect less privileged social strata. The prevalence of unvaccinated children of Asian race/skin color for hepatitis A, was significantly higher, around five times, when compared to White children**.** Although the reasons for ethnic disparities in vaccination coverage are beyond the scope of this work, it can be suggested that Asian-origin families play a role in shaping parents’ attitudes towards the importance of vaccines. This influence includes cultural and religious factors that can directly impact the decision to vaccinate their children.^22^ It is noteworthy that in the past two decades there has been a significant increase in migration flow of people from different origins in Brazil,^23^ and further studies are needed to assess the impact of immigration on vaccine hesitancy in the country.

Non-vaccination against hepatitis A was more common in children whose mothers had paid work. In a predominantly patriarchal society, such as in Brazil, childcare is primarily delegated to mothers.^24^ However, for mothers who have paid jobs, the availability of time to take their children to health centers for routine check-ups and vaccination often does not align with the opening hours of health centers, favoring vaccine hesitancy.^25,26^ Public managers should reconsider opening hours and days of vaccination rooms and other strategies, such as school-based vaccination to mitigate vaccine hesitancy.^27,28^

Children who did not attend daycare centers in the C/D strata showed lower vaccination coverage than those who did. In Brazil, enrollment in daycare centers or nurseries requires presenting an updated vaccination booklet, with records of the vaccines recommended by the PNI.^29^ This public policy is effective and likely has a positive impact on children’s vaccination coverage, especially in the lowest socioeconomic strata.^29^ It not only encourages vaccination, but also contributes to protecting children against vaccine-preventable diseases by ensuring more equitable access to health and education.

Regardless of social stratum, a gradual reduction in hepatitis A vaccination coverage according to birth order was observed. In families with two or more children, it is possible that parents spend less time to the care of the second or subsequent children, and the sense of security provided by the absence of vaccine-preventable diseases in the first-born and their social environment can lead to a lack of concern about complying with the vaccination schedule, resulting in lower vaccination rates for the other children.^24^

The findings of this study show that hepatitis A vaccine coverage is below the desirable target (95%) in all regions of the country. Different socioeconomic factors contribute to non-vaccination in strata A/B and C/D, and must be considered in developing public strategies to reverse the low frequency of vaccinated children, aiming to achieve the vaccination coverage target set by the PNI. The Covid-19 pandemic has further worsened this situation. Administrative data on vaccination coverage after the start of the pandemic are concerning, with hepatitis A vaccination rates reaching 75% in 2020 and 67% in 2021.^30^

Although vaccination coverage is still low, as also evidenced in other countries^9^, the introduction of the hepatitis A vaccine has resulted in a significant reduction in the number of reported cases of the disease, as well as its secondary outcomes (mortality, liver transplant and fulminant hepatitis) in the following years, with an emphasis on children and adolescents.^10^

These advances may regress if significant efforts are not made by managers to strengthen PNI vaccination actions, including improving communication strategies to combat the dissemination of misinformation about the vaccine efficacy and safety.
