# Supplementary material for: Hepatitis A vaccination coverage survey in 24-month-old children living in Brazilian capitals, 2020
Source: Epidemiol Serv Saude. 2024 Oct 21;33(spe2):e20231162. doi: 10.1590/S2237-96222024v33e20231162.en (PMC11493397; doi:10.1590/S2237-96222024v33e20231162.en)
Supplement: Translation

# Translation: Inquérito de cobertura vacinal da hepatite A em crianças de 24 meses de idade residentes em capitais do Brasil, 2020

**DOI:** 10.1590/S2237-96222024v33e20231162.pt

**Authors:** Winny Éveny Alves Moura, Karlla Antonieta Amorim Caetano, Juliana de Oliveira Roque e Lima, Lays Rosa Campos, Grazielle Rosa da Costa e Silva, José Cássio de Moraes, Ana Paula França, Carla Magda Allan Santos Domingues, Maria da Gloria Lima Cruz Teixeira, Sheila Araújo Teles, Grupo ICV 2020, Adriana Ilha da Silva, Alberto Novaes Ramos, Ana Paula França, Andrea de Nazaré Marvão Oliveira, Antonio Fernando Boing, Carla Magda Allan Santos Domingues, Consuelo Silva de Oliveira, Ethel Leonor Noia Maciel, Ione Aquemi Guibu, Isabelle Ribeiro Barbosa Mirabal, Jaqueline Caracas Barbosa, Jaqueline Costa Lima, José Cássio de Moraes, Karin Regina Luhm, Karlla Antonieta Amorim Caetano, Luisa Helena de Oliveira Lima, Maria Bernadete de Cerqueira Antunes, Maria da Gloria Teixeira, Maria Denise de Castro Teixeira, Maria Fernanda de Sousa Oliveira Borges, Rejane Christine de Sousa Queiroz, Ricardo Queiroz Gurgel, Rita Barradas Barata, Roberta Nogueira Calandrini de Azevedo, Sandra Maria do Valle Leone de Oliveira, Sheila Araújo Teles, Silvana Granado Nogueira da Gama, Sotero Serrate Mengue, Taynãna César Simões, Valdir Nascimento, Wildo Navegantes de Araújo

**Affiliations:** 1 Universidade Federal de Goiás, Faculdade de Enfermagem, Goiânia, GO, Brasil; 2 Santa Casa de São Paulo, Faculdade de Ciências Médicas, São Paulo, SP, Brasil; 3 Organização Pan-Americana da Saúde, Brasília, DF, Brazil; 4 Universidade Federal da Bahia, Instituto de Saúde Coletiva, Salvador, BA, Brazil; Universidade Federal do Espírito Santo, Vitória, ES, Brasil; Universidade Federal do Ceará, Departamento de Saúde Comunitária, Fortaleza, CE, Brasil; Faculdade Ciências Médicas Santa Casa de São Paulo, São Paulo, SP, Brasil; Secretaria de Estado da Saúde do Amapá, Macapá, AP, Brasil; Universidade Federal de Santa Catarina, SC, Brasil; Organização Pan-Americana da Saúde, Brasília, DF, Brasil; Instituto Evandro Chagas, Belém, PA, Brasil; Faculdade de Ciências Médicas Santa Casa de São Paulo, Departamento de Saúde Coletiva, São Paulo, SP, Brasil; Universidade Federal do Rio Grande do Norte, Natal, RN, Brasil; Universidade Federal do Ceará, Departamento de Saúde Comunitária, Fortaleza, CE, Brasil; Universidade Federal de Mato Grosso, Cuiabá, MT, Brasil; Universidade Federal do Paraná, Curitiba, PR, Brasil; Universidade Federal de Goiás, Goiânia, GO, Brasil; Universidade Federal do Piauí, Teresina, PI, Brasil; Universidade de Pernambuco, Faculdade de Ciências Médicas, Pernambuco, PE, Brasil; Instituto de Saúde Coletiva, Universidade Federal da Bahia, Salvador, BA, Brasil; Secretaria de Estado da Saúde de Alagoas, Maceió, AL, Brasil; Universidade Federal do Acre, Rio Branco, AC, Brasil; Universidade Federal do Maranhão, Departamento de Saúde Pública, São Luís, MA, Brasil; Universidade Federal de Sergipe, Aracaju, SE, Brasil; Secretaria Municipal de Saúde, Boa Vista, RR, Brasil; Fundação Oswaldo Cruz, Mato Grosso do Sul, Campo Grande, MS, Brasil; Fundação Oswaldo Cruz, Escola Nacional de Saúde Pública Sergio Arouca, Rio de Janeiro, RJ, Brasil; Universidade Federal do Rio Grande do Sul, Porto Alegre, RS, Brasil; Fundação Oswaldo Cruz, Instituto de Pesquisa René Rachou, Belo Horizonte, MG, Brasil; Secretaria de Desenvolvimento Ambiental de Rondônia, Porto Velho, RO, Brasil; Universidade de Brasília, Brasília, DF, Brasil

**Keywords:** Cobertura Vacinal, Programas de Imunização, Vacinas contra Hepatite A, Criança, Fatores Socioeconômicos, Inquéritos Epidemiológicos

## Abstract

**Objetivo:**

Estimar a cobertura vacinal da hepatite A em crianças de 24 meses e identificar fatores associados à ausência de vacinação.

**Métodos:**

Inquérito em amostra estratificada por estratos socioeconômicos em capitais (2020-2022), com estimativa de cobertura e intervalos de confiança de 95% (IC_95_%) e análise de fatores pela razão de prevalência (RP) via regressão de Poisson.

**Resultados:**

Nas 31.001 crianças, a cobertura da hepatite A foi de 88,1% (IC_95_% 86,8;89,2). Nos estratos socioeconômicos (A/B), a variável pais/responsáveis imigrantes foi associada à ausência de vacinação (RP = 1,91; IC_95_% 1,09;3,37); nos estratos C/D, crianças de cor amarela (RP = 4,69; IC_95_% 2,30;9,57), 4ª ordem de nascimento ou mais (RP = 1,68; IC_95_% 1,06;2,66), não frequentar creche/berçário (RP = 1,67; IC_95_% 1,24;2,24) e mãe com trabalho remunerado (RP = 1,42; IC_95_% 1,16;1,74) foram associadas à ausência de vacinação.

**Conclusão:**

Cobertura da hepatite A abaixo da meta (95%), sugerindo-se considerar especificidades dos estratos sociais.

## Contribuições do estudo

**Principais resultados:**

A cobertura vacinal da hepatite A foi de 88%. Ausência de vacinação foi maior em crianças de responsáveis imigrantes (estratos A/B); de cor amarela, 4º filho ou mais, não frequentador de creche/berçário e de mãe com trabalho remunerado (estratos C/D).

**Implicações para os serviços:**

Os resultados deste estudo contribuíram com Ministério da Saúde e Secretarias de Saúde no monitoramento da cobertura vacinal e identificação de fatores que possam impactar negativamente a cobertura vacinal da hepatite A.

**Perspectivas:**

É preciso mais pesquisa sobre o impacto da migração na vacinação contra hepatite A e em geral. Gestores de saúde devem atentar aos diferentes fatores que afetam a vacinação entre os estratos sociais.

## INTRODUÇÃO

Globalmente, estima-se a ocorrência anual de mais de 100 milhões de casos de hepatite A e de 15 mil a 30 mil mortes pela doença, principalmente em países em desenvolvimento, onde o acesso a água tratada e esgotamento sanitário é precário.^1^ No Brasil, as melhorias das condições de vida da população, em especial nas primeiras décadas do século XXI, resultaram na transição epidemiológica da hepatite A, observando-se endemicidade baixa em regiões mais desenvolvidas, como as regiões Sul e Sudeste, e endemicidade intermediária nas regiões menos desenvolvidas, como Centro-Oeste, Norte e Nordeste.^2^

A hepatite A, causada pelo vírus da hepatite A (HAV), vírus RNA da família *Picornaviridae*, gênero Hepatovírus,^1^ é transmitida predominantemente pela via fecal-oral. Em geral, ocasiona doença hepática inflamatória aguda de autorresolução. Raramente evolui para a forma fulminante. Na primeira infância, a infecção em geral é assintomática, aumentando o risco de sintomas com a idade, que incluem febre, mal-estar, fadiga, perda de apetite, diarreia, náuseas, desconforto abdominal, anorexia, mialgia, cefaleia, artralgia e icterícia. Por outro lado, mais de 70% das crianças acima de 5 anos e os adultos são sintomáticos, necessitando de repouso e internação hospitalar. Hepatite A fulminante é um evento raro, e ocorre mais frequentemente em adultos mais velhos.^3^

Vacinas contra hepatite A são seguras e eficazes. Vacinas contra hepatite A inativadas em formaldeído são licenciadas para crianças a partir de 12 meses, por via intramuscular, em duas doses com intervalo mínimo de seis meses entre as doses.^1^ Contudo, estudos em países em desenvolvimento mostraram a efetividade dessas vacinas quando aplicadas em dose única,^4,5^ o que levou a Organização Mundial da Saúde a recomendar os dois esquemas (uma ou duas doses) em crianças.^6^ Assim, considerando o novo perfil endêmico do Brasil, e após análises de custo-efetividade, a Comissão Nacional de Incorporação de Tecnologias no Sistema Único de Saúde recomendou em 2012 a inclusão da vacina contra a hepatite A no calendário básico infantil.^7^ Em 2014, o Programa Nacional de Imunizações (PNI) introduziu essa vacina no calendário infantil, com esquema vacinal de dose única, aos 15 meses de idade.^8^

Um estudo com dados secundários, que avaliou a incidência de hepatite A e a cobertura vacinal da hepatite A no Brasil, após cinco anos de implantação no PNI (2014-2018), constatou que a cobertura vacinal teve o seu pior desempenho em 2014 (60,13%) e melhor desempenho no ano seguinte (97,07%), com variações negativas nos anos posteriores: 71,58% (2016), 82,7% (2017) e 76,72% (2018). A despeito dessa variação, como observado em outros países, houve redução da incidência da hepatite A.^9^

Entre 2014 e 2022, observou-se um decréscimo de mais de 95% na incidência de hepatite A em menores de 10 anos no Brasil,^10^ reforçando a necessidade de se alcançar a meta de cobertura da vacina hepatite A de 95%, recomendada pelo Ministério da Saúde,^11^ para a redução da incidência e o controle da doença. Assim, avaliações periódicas da cobertura vacinal tornam-se imprescindíveis para identificar eventuais falhas a serem superadas pelo serviço, sendo os inquéritos de base populacional as fontes de informação mais confiáveis. O objetivo deste estudo foi estimar a cobertura vacinal da hepatite A em crianças com idade de 24 meses e identificar fatores associados à ausência de vacinação.

## MÉTODOS

Trata-se de um inquérito epidemiológico domiciliar de base populacional que integra o Inquérito Nacional de Cobertura Vacinal (INCV),^12^ realizado nas cinco regiões do Brasil. Esse recorte do estudo avaliou a cobertura da vacina contra hepatite A em crianças nascidas vivas em 2017 e 2018, residentes em 26 capitais e no Distrito Federal, segundo registros do Sistema de Informações sobre Nascidos Vivos (Sinasc). 

A coleta de dados foi realizada entre setembro de 2020 e março de 2022, sendo respeitado o distanciamento físico devido à pandemia de covid-19. 

Informações detalhadas do cálculo amostral e coleta de dados foram descritas em estudo metodológico por Barata et al.^12^ Resumidamente, o tamanho da amostra por inquérito foi calculado considerando os seguintes parâmetros: efeito do desenho de 1,4; prevalência estimada de cobertura vacinal de 70%; erro de estimativa de 5%; e z de 1,96 para intervalo de confiança de 95%. Assim, foram necessárias 452 crianças por inquérito. Considerando-se a heterogeneidade de coberturas vacinais nas capitais, em cada cidade foram realizados de um a quatro inquéritos, dependendo do número de nascidos vivos registrados no Sinasc em 2017 e 2018. Para fins de comparação, o tamanho da amostra foi dividido de forma a se garantir o mesmo número de crianças por estrato socioeconômico. 

Para definição dos estratos socioeconômicos, foram utilizados todos os setores censitários urbanos de cada cidade, segundo as informações do censo demográfico de 2010.^13^ Assim, em cidades onde foi realizado apenas um inquérito, como Porto Velho, o número de crianças por estrato foi 113. Já em cidades como São Paulo, onde foram realizados quatro inquéritos, o número de crianças por estrato foi 452. Para a classificação dos setores, considerou-se renda média dos responsáveis pelo domicílio, proporção de responsáveis alfabetizados e proporção de responsáveis com renda maior ou igual a 20 salários mínimos. Os estratos sociais foram classificados de A a D, com base no nível de consumo das famílias, os pontos de corte estabelecidos pela Associação Brasileira de Empresas de Pesquisa,^14^ critério Brasil: estrato A, alto (42 pontos e mais); estrato B, médio (27 a 41 pontos); estrato C, baixo (16 a 26 pontos); e estrato D, muito baixo (< 16 pontos).

Foram utilizados dois instrumentos de coleta: questionário estruturado com perguntas fechadas e fotografia da caderneta de vacinação. O questionário foi composto por blocos de questões, que para este estudo foram considerados os dados sociodemográficos da criança; dados reprodutivos e sociodemográficos da mãe; dados do domicílio e renda da família; e dados transcritos da caderneta da vacinação (doses válidas aplicadas e data da aplicação) do PNI e do setor privado. Foram consideradas doses válidas (entre 12 e 24 meses) as aplicadas no intervalo e data correta e/ou em intervalos em torno da data prevista. 

A cobertura vacinal foi definida como o percentual de crianças que receberam a vacina contra hepatite A, calculado de acordo com as doses válidas. Para fins deste estudo, considerou-se no numerador o número de crianças nascidas em 2017 e 2018 que receberam a vacina contra hepatite A, e no denominador o total de crianças nascidas vivas no mesmo período nas áreas urbanas das capitais brasileiras. 

A análise das características associadas à ausência de vacinação contra hepatite A nos estratos sociais abrangeu variáveis fundamentais para a compreensão dos fatores subjacentes ao evento. As variáveis associadas à ausência de vacinação contra hepatite A, relativas às crianças, foram sexo (feminino, masculino), ordem de nascimento (número de ordem de nascimento da criança entre os filhos), raça/cor da pele da criança (branca, preta, parda, amarela e indígena), frequentar creche/escola (sim; não) e recebimento da Bolsa Família (sim; não). A variável associada à ausência de vacinação contra hepatite A relativa às famílias foi a renda familiar (expressa em R$: até R$ 1.000,00; de R$ 1.001,00 a R$ 3.000,00; de R$ 3.001,00 até R$ 8.000,00; mais de R$ 8.000,00). As variáveis maternas ou relativas ao responsável foram escolaridade (fundamental I completo ou fundamental incompleto; fundamental II completo ou médio incompleto; ensino médio completo ou superior incompleto; superior completo ou acima), idade no momento do nascimento da criança (< 20 anos; 20 - 34 anos; ≥ 35 anos), raça/cor da pele (branca, preta, parda, amarela e indígena), o exercício de trabalho remunerado (sim; não), a condição de imigrante do responsável (não; sim) e a situação conjugal da mãe (com companheiro estável; sem companheiro estável). 

### Análise dos dados

Conforme descrito detalhadamente por Barata et al.,^12^ por ser uma amostra complexa, pesos amostrais foram calculados para cada domicílio entrevistado, de modo a permitir a estimação sem viés dos parâmetros de interesse da população. Esse procedimento ocorreu em duas etapas: inicialmente foram obtidos pesos amostrais básicos (inverso das probabilidades de inclusão dos domicílios entrevistados), e a seguir, esses pesos foram calibrados para totais populacionais conhecidos. Para tanto, utilizaram-se os dados mais relevantes, ou seja, a vacinação das crianças e os possíveis motivos de ausência de vacinação. O *software* Stata versão 17, módulo *survey data analysis,* foi utilizado para a análise dos dados. A cobertura vacinal da hepatite A geral, por região do Brasil e por estrato social, foi estimada com seus respectivos intervalos de confiança de 95% (IC_95_%). Para identificar potenciais variáveis explanatórias de ausência de vacinação para hepatite A, dois subgrupos foram considerados para análise: estratos A/B e estratos C/D. Inicialmente, análise bivariada foi realizada para identificação de potenciais variáveis associadas à ausência de vacinação da hepatite A. A seguir, as variáveis que apresentaram valor de p < 0,20 foram incluídas em modelos de regressão de Poisson multivariada. A-Link Test foi utilizado para avaliar a qualidade dos modelos finais. Valores de p < 0,05 foram considerados estatisticamente significativos. 

### Aspectos éticos

O inquérito foi aprovado pelos Comitês de Ética em Pesquisa com Seres Humanos do Instituto de Saúde Coletiva da Universidade Federal da Bahia (parecer 3.366.818, em 4/6/2019, Certificado de Apresentação de Apreciação Ética (CAAE) 4306919.5.0000.5030), e da Irmandade da Santa Casa de São Paulo (parecer 4.380.019, de 4/11/2020, CAAE 39412020.0.0000.5479). Antes de iniciar o estudo, os coordenadores locais comunicaram às autoridades de imunização estaduais e municipais sobre a pesquisa. Os entrevistados (pais ou responsáveis) deram consentimento por escrito e permitiram fotografar as cadernetas de vacinação. A coleta de dados ocorreu somente após a autorização dos responsáveis, garantindo a confidencialidade. O banco de dados utilizado excluiu informações de identificação.^12^

## RESULTADOS

Um total de 33.032 crianças foram recrutadas e elegíveis para o estudo e 31.001 crianças participaram do estudo, representando uma perda de 6,15% da amostra inicialmente recrutada. As perdas ocorreram por recusas, insucesso após três tentativas de visita do entrevistador, em horários e dias diferentes, e número previsto de criança, nos conglomerados sorteados inferior ao previsto. A Figura 1 apresenta a cobertura da vacina contra hepatite A na coorte de crianças nascidas em 2017 e 2018, no Brasil e regiões. A cobertura total da vacina hepatite A foi de 88,1% (IC_95_% 86,8;89,2), variando de 86,5% (IC_95_% 84,0;88,7), na região Norte, a 90,7% (IC_95_% 87,793,0), na região Sul. 

**Figura 1:** Cobertura da vacina contra hepatite A em crianças com 24 meses de idade (n = 31.001), residentes nas capitais, de acordo com a região do Brasil, 2020-2021

A Figura 2 apresenta as coberturas da vacina contra hepatite A no Brasil, segundo estrato socioeconômico. Os estratos C (89,4%) e D (88,8%) apresentaram maiores coberturas do que os estratos A (84,6%) e B (85,6%) (p = 0,032). 

**Figura 2:** Cobertura da vacina contra hepatite A em crianças com 24 meses de idade (n = 31.001), residentes nas capitais, segundo estrato socioeconômico, Brasil, 2020-2021

Nos estratos A e B, crianças cujos responsáveis eram imigrantes estrangeiros apresentaram prevalência 1,91 vez maior de ausência de vacinação quando comparadas às que estavam sob os cuidados de responsáveis brasileiros. Por outro lado, em crianças de mães pretas e pardas, as ausências de vacinação foram 66% e 33% menores, respectivamente, comparadas às crianças de mães brancas (Tabela 1).

**Tabela 1:** Frequência, razão de prevalência (RP) bruta e ajustada e intervalo de confiança de 95% (IC_95%_) da ausência de vacinação contra hepatite A em crianças brasileiras de 24 meses (n = 31.001) e suas mães nos estratos sociais A/B, Brasil, 2020-2021

Variável	%	Sim (%)	RP (IC_95_ **%**)^a^	p-valor	RP ajustada (IC_95_ **%**)	p-valor
**Sexo da criança**						
Masculino	14,8	85,2	1,00			
Feminino	14,9	85,1	1,01 (0,74;1,36)	0,966		
**Ordem de nascimento**						
Primeiro	13,4	86,6	1,00		1,00	
Segundo	16,7	83,3	1,25 (0,88;1,76)		1,16 (0,79;1,70)	
Terceiro	15,1	84,9	1,13 (0,71;1,80)	0,159	1,14 (0,78;1,67)	0,115
Quarto ou mais	19,1	80,9	1,43 (0,83;2,46)		1,84 (1,06;3,18)	
**Raça/cor da pele da criança**						
Branca	15,5	84,5	1,00			
Preta	11,7	88,3	0,75 (0,44;1,28)			
Parda	13,8	86,2	0,89 (0,68;1,17)	0,286		
Amarela	7,0	93,0	0,45 (0,13;1,55)			
Indígena	21,3	78,7	1,37 (0,59;3,20)			
**Frequenta creche/escola**						
Sim	13,5	86,5	1,00			
Não	16,3	83,7	1,21 (0,90;1,61)	0,199		
Até 1.000,00	13,8	86,2	1,00			
1.001,00 - 3.000,00	16,9	83,1	1,23 (0,87;1,73)			
3.001,00 - 8.000,00	10,0	90,0	0,72 (0,45;1,18)	0,100		
> 8.000,00	11,1	88,9	0,81 (0,49;1,31)			
**Recebe Bolsa Família**						
Sim	14,1	85,9	1,00			
Não	14,9	85,1	1,06 (0,78;1,45)	0,712		
**Escolaridade da mãe (anos)**						
≤ 8	18,3	81,7	1,00			
9 - 12	14,8	85,2	0,81 (0,44;1,49)			
13 - 15	11,6	88,4	0,64 (0,36;1,12)	0,647		
≥ 16	15,2	84,8	0,83 (0,47;1,45)			
**Idade da mãe (anos) no momento do nascimento da criança**						
< 20	6,7	93,3	1,00			
20 a 34	14,8	85,2	2,20 (0,99;4,90)	0,718		
35 ou mais	14,9	85,1	2,22 (0,99;4,97)			
**Raça/cor da pele da mãe**						
Branca	16,7	83,3	1,00		1,00	
Preta	6,8	93,2	0,40 (0,25;0,67)		0,34 (0,19;0,62)	
Parda	12,0	88,0	0,72 (0,53;0,98)	0,012	0,67 (0,48;0,95)	0,045
Amarela	6,4	93,6	0,38 (0,14;1,02)		0,81 (0,30;2,16)	
Indígena	12,9	87,1	0,77 (0,28-2,11)		0,65 (0,23-1,81)	
**Mãe tem trabalho remunerado**						
Sim	14,7	85,3	1,00			
Não	14,2	85,8	0,97 (0,67;1,40)	0,855		
**Responsável imigrante**						
Não	14,6	85,4	1,00		1,00	
Sim	36,5	63,5	2,49 (1,24;4,99)	0,010	1,91 (1,09;3,37)	0,037
**Situação conjugal**						
Com companheiro	14,1	85,9	1,00			
Sem companheiro	15,0	85,0	1,06 (0,76;1,50)	0,719		

A análise multivariável dos dados das crianças dos estratos C e D revelou que crianças de raça/cor da pele amarela apresentaram prevalência quase cinco vezes maior de ausência de vacinação contra hepatite A comparadas às de raça/cor da pele branca. Observou-se que, à medida que a ordem de nascimento aumentou, como a do segundo filho (RP = 1,31; IC_95_% 1,05;1,64), do terceiro (RP = 1,54; IC_95_% 1,11;2,13) e do quarto filho ou mais (RP = 1,68; IC_95_% 1,06;2,66), as prevalências de ausência de vacinação foram maiores em comparação com as crianças primogênitas. Crianças que não frequentavam creche/berçário e aquelas cujas mães possuíam trabalho remunerado apresentaram, respectivamente, prevalências aumentadas de 1,67 e 1,42, respectivamente, em comparação com aquelas que não possuíam tais características (Tabela 2). 

**Tabela 2:** Frequência, razão de prevalência (RP) bruta e ajustada e intervalo de confiança de 95% (IC_95%_) da ausência de vacinação contra hepatite A em crianças brasileiras de 24 meses (n = 31.001), residentes em 26 capitais e no Distrito Federal, e suas mães, nos estratos sociais C/D, Brasil, 2020-2021

Variável	%	Sim (%)	RP (IC_95_ **%**)^a^	p-valor	RP ajustada (IC_95_ **%**)	p-valor
**Sexo da criança**						
Masculino	10,7	89,3	1,00			
Feminino	11,4	88,6	1,01 (0,86;1,32)	0,574		
**Ordem de nascimento**						
Primeiro	9,5	90,5	1,00		1,00	
Segundo	10,1	89,1	1,14 (0,90;1,44)		1,31 (1,05;1,64)	
Terceiro	12,3	87,7	1,29 (0,94;1,76)	0,002	1,54 (1,11; 2,13)	0,003
Quarto ou mais	16,9	83,1	1,77 (1,22;2,56)		1,68 (1,06;2,66)	
**Raça/cor da pele da criança**						
Branca	9,5	90,5	1,00		1,00	
Preta	11,6	88,4	1,22 (0,87;1,70)		1,14 (0,79;1,65)	
Parda	12,2	87,8	1,28 (1,03;1,59)	0,007	1,03 (0,81;1,31)	
Amarela	35,3	64,7	3,70 (1,87;7,32)		4,69 (2,30;9,57)	0,371
Indígena	7,7	92,3	0,81 (0,28;2,34)		0,57 (0,20;1,65)	
**Frequenta creche/escola**						
Sim	9,1	90,9	1,00		1,00	
Não	12,8	87,2	1,40 (1,09;1,79)	0,008	1,67 (1,24;2,24)	0,001
**Renda familiar (R$)**						
Até 1.000,00	12,2	87,8	1,00			
1.001,00 - 3.000,00	10,1	89,9	0,82 (0,64;1,07)			
3.001,00 - 8.000,00	8,4	91,6	0,69 (0,48;0,99)	0,003		
> 8.000,00	6,1	93,9	0,50 (0,30;0,83)			
**Recebe Bolsa Família**						
Sim	9,9	90,1	1,00			
Não	11,4	88,6	1,15 (0,92;1,44)	0,214		
**Escolaridade da mãe (anos)**						
≤ 8	13,0	87,0	1,00			
9 - 12	13,2	86,8	1,01 (0,74;1,39)			
13 - 15	10,0	90,0	0,77 (0,54;1,10)	0,010		
≥ 16	8,3	91,7	0,64 (0,41;0,98)			
**Idade da mãe (anos) no momento do nascimento da criança**						
< 20 anos	10,8	89,2	1,00			
20 a 34 anos	12,4	87,6	1,15 (0,73;1,81)	0,009		
35 anos ou mais	8,8	91,2	0,81 (0,49;1,34)			
**Raça/cor da pele da mãe**						
Branca	8,7	91,3	1,00		1,00	
Preta	11,7	88,3	0,40 (0,25;0,67)		1,34 (0,97;1,85)	
Parda	12,0	88,0	0,72 (0,53;0,98)		1,37 (1,07;1,76)	
Amarela	13,5	86,5	0,38 (0,14;1,02)	0,012	1,55 (0,59;4,08)	
Indígena	10,1	89,9	0,77 (0,28-2,11)		1,16 (0,46;2,91)	
**Mãe tem trabalho remunerado**						
Sim	11,9	88,1	1,28 (1,05;1,55)	0,012	1,42 (1,16;1,74)	0,001
Não	9,3	90,7	1,00		1,00	
**Responsável imigrante**						
Não	11,0	89,0	1,00			
Sim	17,9	82,1	1,63 (0,87;3,08)	0,128		
**Situação conjugal**						
Com companheiro	10,4	89,6	1,00			
Sem companheiro	11,1	88,9	1,07 (0,84;1,35)	0,576		

## DISCUSSÃO

Esta investigação representa os primeiros dados primários de cobertura vacinal de hepatite A do estudo de base populacional, com análise estratificada por regiões do Brasil e estratos sociais, além de identificar os fatores associados à ausência da vacinação. Todas as regiões do país apresentaram cobertura vacinal da hepatite A abaixo da meta estabelecida pelo Ministério da Saúde, sendo os estratos socioeconômicos mais altos os que apresentaram as menores taxas de cobertura. 

Algumas limitações devem ser consideradas na interpretação dos resultados apresentados. O estudo foi realizado em áreas urbanas e pode não representar as crianças do país, da coorte estudada, embora represente uma parcela significativa. Utilizou-se o Censo Demográfico 2010 para definição dos estratos socioeconômicos, e mudanças urbanas, ao longo dessa década, podem ter ocorrido, mas os dados sociodemográficos do nível familiar ajudaram a mitigar essa limitação. Observou-se uma proporção elevada de mães com idade igual ou superior a 35 anos e nível superior completo, sugerindo viés de seleção. No recorte do presente estudo, idade e escolaridade da mãe não foram associadas a ausência de vacinação contra hepatite A nas análises múltiplas, sugerindo baixo impacto nos resultados. A despeito das limitações, o presente estudo incluiu todas as regiões do país com tamanho amostral robusto e apresenta rigor metodológico. 

A estimativa de cobertura vacinal da hepatite A observada neste estudo foi acima das médias históricas de cobertura dos anos anteriores, sugerindo sub-registro de dados no Sistema de Informação do PNI.^9^ Embora variações regionais tenham sido observadas, as diferenças não foram estatisticamente significativas, ainda que essas coberturas vacinais tenham sido aquém da meta desejável, de 95%, em todas as regiões. Notavelmente, a região Norte, considerada região de endemicidade elevada para hepatite A,^2^ apresentou a menor cobertura vacinal para esta vacina. 

O declínio da cobertura vacinal no Brasil é multifatorial, incluindo mudanças no sistema de registro das vacinas, redução de investimento no setor de saúde e ascensão do movimento antivacina suportado por disseminação de desinformação.^15^ O cenário atual foi impactado pelo desabastecimento ocasional de imunizantes, decorrente de dificuldades na produção, e por desafios operacionais que afetaram a execução adequada dos programas de vacinação.^16^

Os estratos sociais A/B, mais favorecidos economicamente, apresentaram coberturas vacinais contra hepatite A inferiores àquelas dos estratos C/D, menos favorecidos. Acredita-se que as altas coberturas vacinais do PNI até meados da década de 2010, reduziram/eliminaram várias infecções infantis, e podem ter levado a uma falsa sensação de segurança dos pais em relação à saúde de seus filhos.^17^ O aumento da adesão ao movimento antivacina nas classes mais privilegiadas têm contribuído para a redução das taxas de vacinação dentro desses estratos.^18^

O aumento da cobertura vacinal nas classes menos privilegiadas economicamente pode ser atribuído, principalmente, à abrangente atuação da Estratégia Saúde da Família (ESF) e ao acesso facilitado aos estabelecimentos de saúde. Essa iniciativa expandiu significativamente a oferta de medidas preventivas, com foco especial nas comunidades mais vulneráveis.^17^ As equipes da ESF desempenham um papel ativo no acompanhamento das famílias, monitorando a saúde das crianças, fornecendo orientações sobre a importância da imunização e garantindo a adesão ao calendário de vacinação.^19^ O Programa Bolsa Família, uma estratégia fundamental na transferência de renda, desempenha um papel ímpar na promoção da saúde infantil e na garantia da vacinação, ao estabelecer condicionalidades de saúde que incluem a atualização do calendário de vacinação.^20^

Analisou-se o efeito de possíveis características sociodemográficas na baixa cobertura vacinal da hepatite A, considerando-se em conjunto os estratos sociais A/B e C/D. 

Para os estratos A/B, a cor da criança não influenciou na cobertura vacinal da hepatite A, mas a cor da mãe exerceu um papel protetor. Crianças de mães pretas e pardas apresentaram prevalências de ausência de vacinação 66% e 33%, respectivamente, menores de vacinação comparadas às de mães brancas. Contudo, deve-se ressaltar que, dentro desses estratos, observa-se uma diferença na distribuição de renda entre as famílias de crianças de mães pretas e pardas e mães brancas. Uma proporção menor de famílias com mães de raça/cor da pele preta e parda (13,6%) tinha renda superior a R$ 8.000,00 em comparação com as famílias de mães brancas (44,8%) (dados não apresentados), corroborando os achados de melhores coberturas vacinais em famílias de menor renda. 

Observou-se nos estratos A/B que ser criança sob a responsabilidade de imigrantes foi um fator associado à ausência de vacinação contra hepatite A. Essa relutância em relação à vacinação entre os imigrantes pode ser atribuída a vários fatores, como o receio e a desinformação sobre possíveis efeitos adversos das vacinas, falta de conhecimento sobre as doenças, desconfiança nos sistemas de saúde dos países anfitriões, barreiras linguísticas e influência das crenças e da mídia de seus países de origem.^21^

Em consonância com os achados nos estratos A/B, questões migratórias parecem perpassar também os estratos sociais menos favorecidos. A prevalência de crianças de raça/cor da pele amarela não vacinadas para hepatite A foi significativamente maior, cerca de cinco vezes, quando comparadas às de cor branca**.** Embora as razões para as disparidades étnicas na cobertura vacinal fujam do escopo deste trabalho, pode-se sugerir que famílias de origem asiática desempenham um papel na moldagem das atitudes dos pais em relação à importância das vacinas. Essa influência inclui fatores culturais e religiosos que podem impactar diretamente a decisão de vacinar suas crianças.^22^ Deve-se ressaltar que, nas últimas duas décadas, houve aumento significativo do fluxo migratório de pessoas de várias origens no Brasil,^23^ sendo desejável estudos adicionais para avaliar o impacto da imigração na hesitação vacinal no país.

A ausência de vacinação contra hepatite A foi mais frequente em crianças de mães com trabalho remunerado. Em uma sociedade predominantemente patriarcal, como no Brasil, o cuidado dos filhos é majoritariamente delegado às mães.^24^ No entanto, para as mães que possuem trabalhos remunerados, a disponibilidade de horário para levar os filhos às unidades de saúde para consultas de rotina e vacinação, muitas vezes, não converge com os horários de funcionamentos das unidades de saúde, favorecendo a hesitação vacinal.^25,26^ Gestores públicos deveriam reavaliar horários e dias de funcionamento das salas de vacina e outras estratégias, como a vacinação no ambiente escolar, para mitigar a hesitação vacinal.^27,28^

Crianças que não frequentavam creches nos estratos C/D apresentaram menor cobertura vacinal do que as que frequentavam. No Brasil, a matrícula em creches ou berçários requer a apresentação da caderneta de vacinação atualizada, com os registros das vacinas recomendadas pelo PNI. ^29^ Essa política pública é eficaz e muito provavelmente tem um impacto positivo na cobertura vacinal das crianças, especialmente nos estratos socioeconômicos mais baixos.^29^ Ela não apenas incentiva a vacinação, mas contribui para a proteção das crianças contra doenças evitáveis por meio da imunização, garantindo um acesso mais igualitário à saúde e à educação.

Independentemente do estrato social, observou-se uma redução gradativa na cobertura vacinal da hepatite A de acordo com a ordem de nascimento. Em famílias com dois ou mais filhos, é possível que os pais dediquem menos tempo no cuidado ao segundo ou aos filhos subsequentes, e a sensação de segurança proporcionada pela ausência de doenças imunopreveníveis no primogênito e em seu entorno social pode favorecer despreocupação em relação ao cumprimento do calendário vacinal, resultando em taxas de vacinação mais baixas para os demais filhos.^24^

Os achados deste estudo mostram que a cobertura da vacina contra hepatite A está abaixo da meta desejável (95%) em todas as regiões do país. Fatores socioeconômicos distintos contribuem para a ausência de vacinação nos estratos A/B e C/D, e devem ser considerados na construção de estratégias públicas que revertam a baixa frequência de crianças vacinadas, visando ao alcance da meta de cobertura vacinal estabelecida pelo PNI. A pandemia de covid-19 agravou ainda mais essa situação. Os dados administrativos de cobertura vacinal após o início da pandemia são preocupantes, com taxas da vacina contra hepatite A atingindo 75% em 2020 e 67% em 2021.^30^

Apesar de a cobertura vacinal ainda ser baixa, como também evidenciado em outros países,^9^ a introdução da vacina contra hepatite A resultou em uma redução significativa no número de casos notificados da doença, bem como de seus desfechos secundários (mortalidade, transplantes hepáticos e hepatite fulminante) nos anos seguintes, com ênfase em crianças e adolescentes.^10^

Tais avanços podem retroceder, caso não haja esforço significativo dos gestores para fortalecer as ações de vacinação do PNI, incluindo uma melhoria das estratégias de comunicação para combater à disseminação de informações errôneas sobre a eficácia e segurança das vacinas.
